# P2 Manifests Subjective Evaluation of Reward Processing Under Social Comparison

**DOI:** 10.3389/fpsyg.2022.817529

**Published:** 2022-02-18

**Authors:** Feng Zou, Xiaoya Li, Fenfang Chen, Yao Wang, Li Wang, Yufeng Wang, Xin Wu, Meng Zhang

**Affiliations:** ^1^Department of Psychology, Xinxiang Medical University, Xinxiang, China; ^2^Department of Education, Hangzhou Normal University, Hangzhou, China

**Keywords:** subjective evaluation, social comparison, reward evaluation, P2, LPP

## Abstract

Several recent studies have found that when the other’s gain is greater, even subjects’ reward may seem like a loss and lead to a negative experience. These studies indicate the complexity of reward evaluation in the context of social comparison. The satisfaction rating of reward outcome not only depends on objective social comparison but also on subjective evaluation. However, less is known about the neural time course of subjective evaluation. Therefore, we employed a 2 (subjective evaluation: advantageous vs. disadvantageous) × 2 (comparison direction: upward vs. downward) within-subjects factorial design, in which we manipulated the reward distribution for the subjects. Electroencephalography (EEG) responses were recorded, while two subjects concurrently but independently performed a simple dot-estimation task that entailed monetary rewards. Behavioral results showed that the subjects were more satisfied with the advantageous distribution, regardless of upward or downward comparison. The analysis of event-related potentials (ERPs) revealed that disadvantageous distribution elicited a larger P2 than advantageous distribution, and this effect was not modulated by comparison direction. In contrast, the late positive potential (LPP) showed an effect of comparison direction independent of subjective evaluation. The data suggest that subjective evaluation acts upon the early stage of reward processing and manifests in the P2 component, whereas social comparison plays a role in the later appraisal process.

## Introduction

Social comparison is the process of thinking about the information of one or more other people in relation to the self (Wood, 1996). In daily life, social comparison is ubiquitous and plays a crucial role in human adaptation and survival. For example, people may make an upward comparison (compared with a superior other) hoping to improve their abilities (Collins, 1996) or make a downward comparison (compared with an inferior other) to increase self-evaluations (Wills, 1981). In recent years, many studies have concentrated on the influence of social comparison on reward processing. Social psychologists have shown that the subjective wellbeing of people not only depends on absolute income but also on relative income (Smith et al., 1989; Hagerty, 2000). Fliessbach et al. (2007) explored the impact of social comparisons on reward processing using functional MRI. They found that activity in the ventral striatum, a reward-related brain structure, is affected by the variation in the comparison of payments of the participants (Fliessbach et al., 2007). This finding was verified by other studies using slightly different paradigms, suggesting an immediate impact of social comparison on ventral striatal responses (Dvash et al., 2010; Bault et al., 2011; Dohmen et al., 2011). These studies from the perspectives of behavior and brain mechanisms highlight the importance of studying reward processing in the context of social comparison.

With the advantage of high time resolution of electroencephalography (EEG) technology, previous research has identified that social comparison may affect outcome event-related potential (ERP) components, including the P2, the feedback-related negativity (FRN), the P300, and the late positive potential (LPP) (Qiu et al., 2010; Wu et al., 2012; Kedia et al., 2014; Luo et al., 2015). The first P2 component is a positive, fronto-central ERP component, peaking around 150–250 ms post-feedback onset (Hillyard et al., 1973; Salim et al., 2015). A recent study suggested that the brain processes social comparison information at an early stage of outcome evaluation, with a larger P2 amplitude when subjects faced outcomes that were different from others (Luo et al., 2015). However, several other related studies did not find this component (Qiu et al., 2010; Wu et al., 2012). Consistent with the reward processing domain, the P2 component is thought to reflect attention selection and salience detection (Carretié et al., 2005; Potts et al., 2006; Bellebaum et al., 2010; San Martín et al., 2010; Xu et al., 2011; Schuermann et al., 2012; Flores et al., 2015; Glazer et al., 2018; Wischnewski and Schutter, 2018, 2019).

A second ERP component related to social comparison is the FRN, which is a fronto-central negative deflection, peaking around 250 ms post-feedback onset. Traditionally, the FRN is thought to code a reward prediction error in the human brain and reflects the evaluation of outcome along a good-bad dimension (Holroyd and Coles, 2002; Hajcak et al., 2007; Sambrook and Goslin, 2015). Several ERP studies tested whether social comparison modulated the FRN. For example, using a simple time-estimation reaction-time task, Boksem et al. (2011) found that enhanced FRN was elicited when the outcomes were worse than those of another player (Boksem et al., 2011). However, two studies employing similar experimental designs did not find FRN modulations related to social comparison (Qiu et al., 2010; Wu et al., 2012). These inconsistent results may be because the FRN is modulated by several factors, such as outcome probability, expectations, and perceived task difficulty, which vary with experimental paradigm changes (Kedia et al., 2014).

A third ERP component is the P300, which is a centro-parietal positive-going deflection, peaking from 300 to 600 ms after feedback onset. Some studies have shown that the maximum period of the P300 component may be extended to approximately 800 ms (Bonnefond and der Henst, 2009; Li et al., 2014). Previously, the P300, which was frequently reported in the reward processing, was generally considered to be related to the motivationally significant or relevant stimuli that capture attentional resources (Yeung and Sanfey, 2004; Hajcak et al., 2005; Nieuwenhuis et al., 2005; Sato et al., 2005; Wu and Zhou, 2009; Zhou et al., 2010). So far, only one study found that the P300 component was modulated by the social comparison direction, not the feedback valence (Wu et al., 2012).

Finally, the LPP is a positive-going centro-parietal ERP component starting around 500 ms after feedback onset and lasts for several hundred milliseconds (Cuthbert et al., 2000; Schupp et al., 2006; Hajcak et al., 2009). In the context of reward processing, previous studies often conflate the P300 and LPP measurements because of their strong temporal overlap, but less is known about the functional differences between P3 and LPP in outcome processing (see review, Glazer et al., 2018). Whereas the LPP in the social comparison situations was often observed and modulated by comparison direction, the more unequally, the larger the LPP amplitude detailed (Qiu et al., 2010; Wu et al., 2011; Luo et al., 2015).

Previous research has shown that upward comparison elicits negative emotional responses and downward comparison elicits positive emotional reactions (Tesser and Collins, 1988; Aspinwall and Taylor, 1993; White et al., 2006; Dvash et al., 2010). However, in reality, individuals often encounter more than one comparison object, and the degree of difference between individual and comparison object is also diverse. In this study, Du and colleagues (2013) examined satisfaction with outcomes in a three-person comparison. In their experiment, one subject and two pseudo-subjects completed a simple number estimation task and received different monetary rewards. Interestingly, behavioral results suggested that despite the two reward distributions representing an upward comparison, the subjects were more satisfied with one distribution scheme (e.g., subject/pseudo-A/pseudo-B = 20/40/60) than the other distribution scheme (e.g., subject/pseudo-A/pseudo-B = 20/50/50). This finding shows that satisfaction rating seems to be influenced by subjective evaluation besides comparison direction. Specifically, in the upward comparison context, comparison objects with small differences may be evaluated as subjectively advantageous stimuli compared to comparison objects with large differences, which can improve individual satisfaction to some extent. Consistent with this point of view, another experiment found that even when subjects won money, they expressed envy if another player won more money. In contrast, when they lost money, they expressed joy and schadenfreude if another player lost more money (Dvash et al., 2010). Despite this phenomenon being more in line with actual life experiences of the people, the underlying neurobiological substrate of this social comparison process is not well understood.

With EEG recording, the primary objective of this study was to explore how subjective evaluation moderates the satisfaction rating of reward outcome in the context of social comparison and how the brain responds to such modulation. To this end, we revised the simple dot-estimation task used by Fliessbach et al. (2007). Two subjects concurrently but independently performed a simple dot-estimation task and received the monetary reward on each trial. The experiment employed a 2 (subjective evaluation: advantageous vs. disadvantageous) × 2 (comparison direction: upward vs. downward) within-subject factorial design, in which we manipulated the reward distribution for the subjects. Reward conditions are illustrated in Table 1. Locke (1976) proposed that satisfaction is driven by the congruence between what we obtain and what we value (Locke, 1976). In this sense, people are relatively satisfied with these two kinds of reward distribution: they all get rewards, but they get more than others (i.e., self/others: 40/10 vs. 20/10), or although they get less than others, the difference is not too much (i.e., self/others: 10/20 vs. 10/40). Therefore, the two reward distributions are defined as subjectively advantageous conditions. In contrast, the other two reward distributions (i.e., self/others: 20/10 and 10/40) are defined as subjectively disadvantageous conditions. According to the previous neurophysiological studies, evaluation of reward outcome can be divided broadly into two related processes: first, an early evaluation of the motivational/affective significance of the reward feedback stimuli, which may manifest in the P2 or FRN component, followed by a more elaborative evaluation, which may manifest in the P300 or LPP (Goyer et al., 2008; Leng and Zhou, 2010; Wu et al., 2012). We anticipate that subjective evaluation as a factor would be processed at the abovementioned stages. However, due to the confounding of relevant research findings and the limited evidence for direct research on subjective evaluation, it is unlikely to make the formation of an accurate prediction model extremely.

**TABLE 1:** Reward conditions in the experiment.

	Upward	Downward	Fillers
**Advantageous**	10–20	40–10	0–0 0–10/10–0
**Disadvantageous**	10–40	20–10	10–10/40–40

*There are four conditions of interest: advantageous upward (labeled “AU” below), advantageous downward (“AD”), disadvantageous upward (“DU”), and disadvantageous downward (“DD”). When both subjects completed the task correctly, one of four possible reward conditions (AU, AD, DU, and DD) was randomly selected. For example, the two numbers ra0–20numbers randomly selected. For-upward comparison context, one subject received 10 ¥, another player received 20 ¥. Meanwhile, to establish a more realistic experiment, we set the filling conditions, in which at least one of the two subjects was incorrect, and both subjects were correct but received the same amount of money.*

## Materials and Methods

### Participants

Forty-eight healthy students (24 women; mean age 18.48 ± 0.90 years) from Xinxiang Medical University participated in the experiment. Two subjects had to be excluded from the analysis due to excessive ocular artifacts, so that data from 46 subjects were finally analyzed. All subjects were right-handed and had normal or corrected-to-normal vision. None of them had a history of neurological or psychiatric problems. All subjects gave written informed consent in accordance with the Declaration of Helsinki. Furthermore, for subjects under 18 years of age, we obtained the written informed consent of their parents. The Institutional Ethics Committee of Xinxiang Medical University approved the study protocol.

### Procedure

Each round of the experiment includes two subjects of the same gender to control the potential impact of gender differences in reward processing (Fliessbach et al., 2007; Warthen et al., 2020). Before the experiment, both subjects were asked that they would sit in two adjacent cabins to finish a simple number estimation task simultaneously through the computer network. They did not know each other and had no chance to communicate before the end of the experiment. They were also informed that the computer would allocate monetary rewards based on their performance in the task (i.e., response accuracy and response time). Unknown to the subjects, their performance (whether the estimates were right or wrong) and reward distribution were presented in a pseudo-randomized and counterbalanced way.

From the perspective of the subjects, reward feedback could be either upward comparison (when the subject got less money than the other player) or downward comparison (when the subject got more money than the other player). During upward comparison conditions, the reward feedback could be subjectively advantageous (reward distribution with a small difference, i.e., 10–20) or subjectively disadvantageous (reward distribution with a large difference, i.e., 10–40). In contrast, during downward comparison conditions, reward distribution with a small difference (i.e., 20–10) represents a subjectively disadvantageous condition, but reward distribution with a large difference (i.e., 40–10) represents a subjectively advantageous condition. There are four conditions of interest (refer to Table 1), namely, advantageous upward (labeled “AU” below), advantageous downward (“AD”), disadvantageous upward (“DU”), and disadvantageous downward (“DD”). When both subjects completed the task correctly, one of the four reward conditions (i.e., AU, AD, DU, and DD) was randomly selected. Meanwhile, to establish a more realistic experiment, we set the filling conditions, in which at least one of the two subjects was incorrect, and both subjects were correct but received the same amount of money.

**FIGURE 1:**
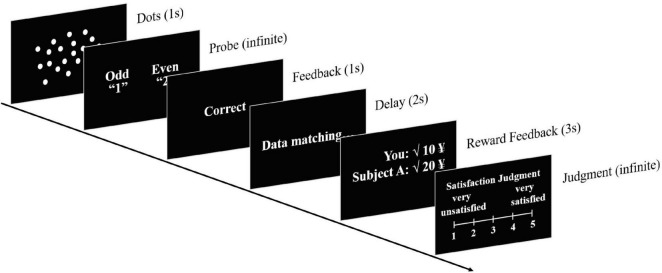
A sequence of events in a single trial.

Each trial began with a screen with a varying number of white dots (10–50) for 1,000 ms (refer to Figure 1). Subsequently, subjects had to judge the number of dots as “odd” or “even” by pressing the key “1” or “2,” respectively. The presentation stopped when subjects had responded. Then, a screen presented “Correct” or “Incorrect” for 1,000 ms as response feedback. The phrase “data matching” was presented for another 2,000 ms, implying that the computer was doing background processing in line with the performance of both subjects. Next, a reward feedback screen was displayed for 3,000 ms. This screen showed both players whether they were correct or not (indicated by a tick or a cross), together with the respective monetary rewards in this trial. Finally, subjects were required to rate their satisfaction with the reward feedback by pressing one of the numeric keys 1–5 (1 representing “very unsatisfied” and 5 representing “very satisfied”). The next trial started after a time interval of 250 ms.

During the estimation task, the subject was seated comfortably about 1 m from a 19-in CRT display. The experiment was coded and performed using E-Prime 2.0 software (Psychology Software Tools, Pittsburgh, PA, United States). One of the four experimental conditions (AU/AD/DU/DD) had 35 trials. In addition, another 112 trials, corresponding to the filling conditions, were used as fillers. The 252 trials were randomly mixed and were divided in equal numbers into two blocks. Subjects could take a rest between the two blocks. At the end of the experiment, each subject was paid and thanked for their participation.

### Electroencephalography Recording and Analysis

Continuous EEG was acquired using Neuroscan (Compumedics, Charlotte, United States) SynAmps2 64-channel amplifier and an Ag/AgCl Electro-Cap according to the standard international 10–20 system. A cephalic (forehead) location was used for the ground, and the left mastoid was chosen for reference. Vertical electrooculogram (EOG) was recorded from electrodes placed above and below the left eye. Horizontal EOG was recorded from electrodes placed at the left and right orbital rim. All interelectrode impedance was kept below 5 kΩ. The EEG signal was amplified with a band-pass filter from 0.01 to 100 Hz and digitized online at a sampling rate of 500 Hz.

The EEGLAB toolbox (Makeig et al., 2004) under the MATLAB environment was used for offline analysis. Data were re-referenced to the mean of the left and right mastoids and were digitally filtered from 0.1 to 30 Hz. Epochs were extracted from 0.2 s before to 1 s after the onset of the stimulus of interest. After baseline correction (-0.2 to 0 s), ocular artifacts were rejected by the independent component analysis. Trials with amplitudes exceeding ± 80 μV at any electrode were excluded from further analysis.

**FIGURE 2:**
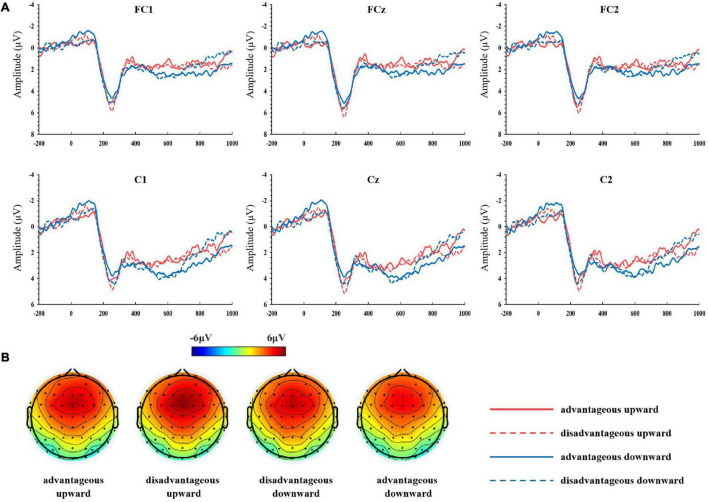
**(A)** Grand-averaged event-related potentials (ERPs) at FC1, FCz, FC2, C1, Cz, and C2 electrodes for P200 with a comparison of the outcome under various feedback conditions. **(B)** Topographical voltage distributions of four conditions of interest in the time range of the P2 (230–270 ms).

**FIGURE 3:**
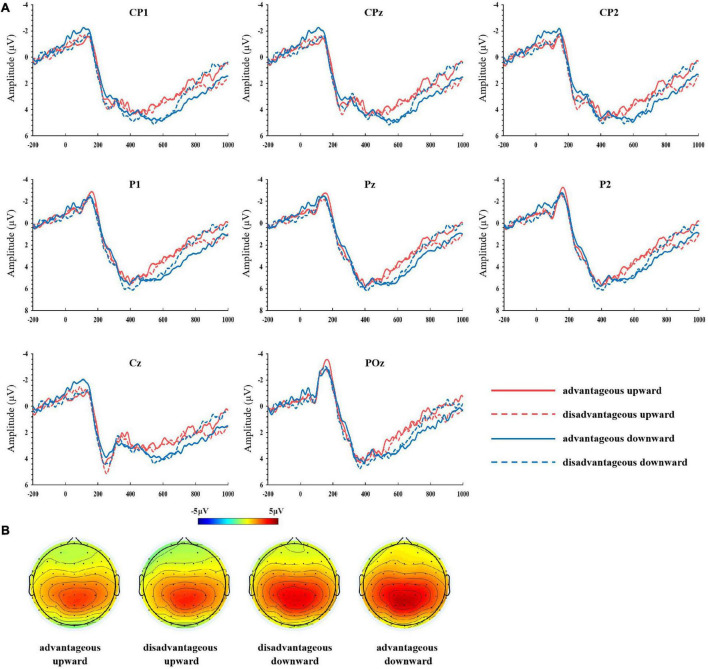
**(A)** Grand-averaged ERPs at Cp1, Cpz, Cp2, P1, Pz, P2, Cz, and POz electrodes for late positive potential (LPP) with a comparison of the outcome under various feedback conditions. **(B)** Topographical voltage distributions of four conditions of interest in the time range of the LPP (500–800 ms).

The ERP components analyzed included P2, FRN, P300, and LPP. Based on previous studies (Gehring and Willoughby, 2002; Yeung and Sanfey, 2004; Wu et al., 2012; Luo et al., 2015; Pornpattananangkul and Nusslock, 2015; Kwak et al., 2019) and topographical maps (refer to Figures 2, 3), the FC1, FCz, FC2, C1, Cz, and C2 electrodes were included in the calculations of the P2 component; for the FRN, one classical electrode, FCz, was included; for the P300, one classical electrode, Pz, was included; for the LPP, Cz, Cp1, Cpz, Cp2, P1, Pz, P2, and POz electrodes were included. We chose 230–270, 320–400, 350–450, and 500–800 ms after the onset of reward stimulus for analyzing P2, FRN, P300, and LPP. These time windows were selected according to visual inspection of waveforms and the classical definitions for these components.

Analysis of variance was conducted with three within-subject factors, namely, subjective evaluation (advantageous vs. disadvantageous), comparison direction (upward vs. downward), and electrode. Simple effect analysis was conducted when the interaction effect was significant. The Greenhouse–Geisser correction was used in all statistical analyses whenever appropriate. The Bonferroni correction was applied for multiple comparisons.

## Results

### Behavioral Results

**FIGURE 4:**
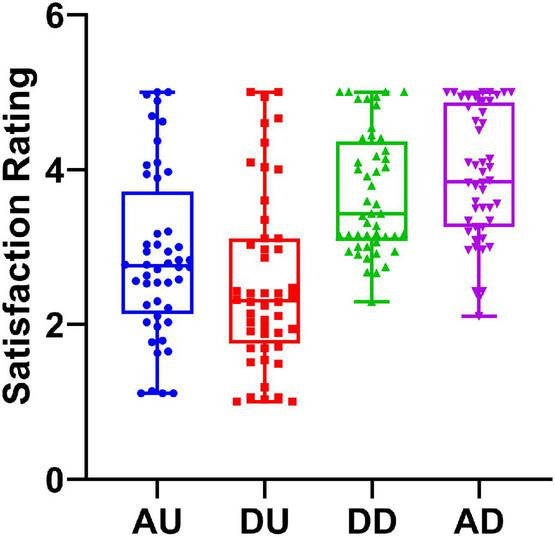
Satisfaction rating under various feedback conditions. Advantageous upward condition is shown in blue; disadvantageous upward condition is shown in red; disadvantageous downward condition is shown in green; and advantageous downward condition is shown in purple.

A 2 (subjective evaluation: advantageous vs. disadvantageous) × 2 (comparison direction: upward vs. downward) repeated-measures ANOVA revealed a significant main effect of comparison direction, *F*(1, 45) = 46.22, *p* < 0.001, η^2^ = 0.507. Pairwise comparison showed that subjects were more satisfied with downward comparison (mean ± *SD*, 3.80 ± 0.82) than with upward comparison (2.69 ± 1.11). The main effect of subjective evaluation was significant, *F*(1, 45) = 47.61, *p* < 0.001, η^2^ = 0.514. Pairwise comparison showed that a satisfaction rate for advantageous distribution (3.39 ± 1.11) was higher than that for disadvantageous distribution (3.10 ± 1.13). However, the comparison direction × subjective evaluation interaction effect was not statistically significant, *F*(1, 45) = 0.94, *p* = 0.338, η^2^ = 0.020. The behavioral result is presented in Figure 4.

### Event-Related Potential Results

Analysis of the P2 component revealed a significant main effect for the subjective evaluation factor, *F*(1, 45) = 4.17, *p* < 0.05, η^2^ = 0.085. Pairwise comparison showed that the disadvantageous distribution (mean ± *SD*, 4.93 ± 0.44 μV) elicited a larger P2 than the advantageous distribution (4.36 ± 0.41 μV). The main effect of comparison direction factor was not significant, *F*(1, 45) = 1.59, *p* = 0.214, η^2^ = 0.034. No significant interaction of comparison direction × subjective evaluation was found, *F*(1, 45) = 0.24, *p* = 0.625, η^2^ = 0.005 (refer to Figure 2A).

No significant effects were observed for the FRN component (subjective evaluation: *F*(1, 45) = 0.001, *p* = 0.977, η^2^ < 0.001; comparison direction: *F*(1, 45) = 3.541, *p* = 0.066, η^2^ = 0.073; subjective evaluation × comparison direction: *F*(1, 45) = 0.588, *p* = 0.447, η^2^ = 0.013), indicating that the FRN was similar across AU, AD, DU, and DD comparison conditions.

No significant effects were observed for the P300 component (subjective evaluation: *F*(1, 45) = 1.305, *p* = 0.259, η^2^ = 0.028; comparison direction: *F*(1, 45) = 1.12, *p* = 0.296, η^2^ = 0.024; subjective evaluation × comparison direction: *F*(1, 45) = 0.317, *p* = 0.576, η^2^ = 0.007), indicating that the P300 was similar across AU, AD, DU, and DD comparison conditions.

Analysis of the LPP component revealed a significant main effect for the comparison direction factor, *F*(1,45) = 23.27, *p* < 0.001, η^2^ = 0.341. Pairwise comparison showed that the LPP was larger in response to downward comparison (3.88 ± 0.37 μV) than upward comparison (2.97 ± 0.30 μV). Neither the main effect of the subjective evaluation factor, *F*(1, 45) = 0.10, *p* = 0.750, η^2^ = 0.002, nor the interaction of comparison direction × subjective evaluation, *F*(1, 45) = 1.59, *p* = 0.214, η^2^ = 0.034, was significant (refer to Figure 3A).

## Discussion

This study investigated the influence of subjective evaluation and comparison direction on reward processing under social comparison contexts. Consistent with previous studies (Dvash et al., 2010; San Martín et al., 2010; Du et al., 2013), we found that the subjects were more satisfied with reward distribution when they received more than other players. Furthermore, our results showed that the satisfaction rating was modulated by subjective evaluation. Specifically, the subjects were more satisfied with the advantageous distribution, regardless of upward or downward comparison. Electrophysiologically, we observed a subjective evaluation effect on the P2, and this effect was not modulated by comparison direction. In contrast, the LPP component displayed the modulation effect of comparison direction but was not sensitive to the subjective evaluation factor. However, the FRN and P300 components were neither sensitive to subjective evaluation nor comparison direction.

The early P2 component displayed a differential processing of advantageous and disadvantageous outcomes. Results show that the P2 amplitude was larger in response to disadvantageous distribution as compared to advantageous distribution. This finding suggests that the brain differentially processes advantageous and disadvantageous outcomes already at an early stage. Previous studies on the effects of valence have yielded rather inconclusive, and even conflicting, conclusions (Carretié et al., 2005; Bellebaum et al., 2010; Xu et al., 2011; Schuermann et al., 2012). Our findings support the view that the P2 is an arousal component that reflects a valence effect associated with feedback (Wischnewski and Schutter, 2018). It should be noted that most previous studies did not consider subjective evaluation as a variable factor. Indeed, the variable subjective evaluation represents the subjective judgment of an individual on the value of feedback outcome, which is not exactly the same as the valence concept involved in other studies (e.g., win/loss and positive/negative). Therefore, it may not be appropriate to directly compare the results of our experiment with those of other relevant studies. In this study, the modulation effect of P2 by subjective evaluation suggests that P2 may serve as a neural index for the binary classification of the advantageous and disadvantageous outcome. The P2 component plays a role in salience detection in the early stage of reward processing; in this sense, more attention was given to disadvantageous feedback.

The P2 amplitude was not modulated by comparison direction, a result inconsistent with those observed in a recent study on outcome evaluation (Luo et al., 2015). Their experiment employed a three-person comparison paradigm, in which researchers asked the subjects to play a lottery game with two pseudo-players simultaneously, and presented them with their outcome and those of two other players. The comparison between self and others showed hierarchical characteristics. They supposed that at the early stage of outcome evaluation, the processing of social comparison information was relatively rough, and the detailed information was not yet processed until later stages.

Unlike the P2, we found that the LPP component was modulated by social comparison. The LPP amplitude was larger when the subject received a monetary reward more than another player. The results are generally consistent with previous studies on outcome evaluation (Qiu et al., 2010; Wu et al., 2011, 2012). Traditionally, the LPP is thought to reflect sustained attention and extended cognitive processing of the motivationally salient, high-arousal stimuli (Schupp et al., 2000; Groen et al., 2008; Dunning and Hajcak, 2009; Althaus et al., 2010; Pornpattananangkul and Nusslock, 2015). In this study, reward feedback presented only one valence category, “gain,” and the difference between the conditions was whether the subject received a monetary reward more or less than the other player. From a motivational perspective, the downward comparison conditions (e.g., self/others: 20/10 and 40/10) are evaluated as most motivational relevant in comparison with the upward comparison conditions (e.g., self/others: 10/20 and 10/40). Thus, we interpret this LPP effect as reflecting motivationally salient outcomes.

Moreover, our results did not find the FRN effect and P300 effect, which is inconsistent with many previous studies (Hajcak et al., 2005, 2007; Bellebaum et al., 2010; Kreussel et al., 2012). According to the independent coding model proposed by Yeung and Sanfey (2004), two key reward features, namely, valence (i.e., loss vs. gains) and magnitude (i.e., large vs. small), are encoded separately in the brain, with the FRN being sensitive to reward valence and the P3 to reward magnitude. However, other studies have shown that FRN and P300 are all affected by outcome probability, with unexpected outcomes eliciting larger amplitude (Campbell et al., 1979; Horst et al., 1980; Gibson et al., 2006; Hajcak et al., 2007; Goyer et al., 2008; Jessup et al., 2010; Alexander and Brown, 2011; Kreussel et al., 2012; San Martín, 2012; Talmi et al., 2013; Watts et al., 2017). Consistent with this point of view, in this study, the conditions of interest that had equal trials were presented in a pseudo-random manner. Therefore, subjects were not able to form a reliable reward prediction for a particular parity judgment and thus could not detect prediction errors. This may explain why FRN and P300 were not affected by subjective evaluation and comparison direction. However, it should be noted that the main effect of the comparison direction in this study was marginally significant. Therefore, researchers should be very cautious when interpreting the results.

## Conclusion and Limitations

To sum up, this study tentatively distinguishes subjective evaluation from objective social comparison. Behavioral results reveal the complexity of social comparison in the process of reward evaluation, that is, the satisfaction rating of reward outcome not only depends on social comparison but also on subjective evaluation. EEG results suggest that subjective evaluation acted upon earlier processing stage and manifested in the P2 component. This finding highlights the importance of P2 in reward processing and further extends the significance of the early ERP components of reward processing. Furthermore, in terms of theory, the strong temporal overlap among reward-related components (Glazer et al., 2018) and the complexity of social comparison itself lead to substantial conflict in the existing literature. Independent coding theory proposes that the two attributes of reward stimulus, i.e., valence and magnitude, are processed independently in the early and late stages of reward outcome processing, which are reflected in FRN and P300, respectively. However, this study showed that subjective evaluation also affected reward processing, as reflected in the P2, whereas the effect of comparison direction on reward processing was mainly in the later stage of outcome evaluation, as reflected in the LPP. This extends the theory to some extent. In the future, more research is needed to clarify how humans subjectively process feedback outcomes and what role these classical components play in this process. Moreover, given the complexity of social comparison and the flexibility of individuals, it is necessary to explain individual differences in future studies of social comparison.

## Data Availability Statement

The original contributions presented in the study are included in the article/supplementary material, further inquiries can be directed to the corresponding author/s.

## Ethics Statement

The studies involving human participants were reviewed and approved by the Institutional Ethics Committee of Xinxiang Medical University. Written informed consent to participate in this study was provided by the participants’ legal guardian/next of kin.

## Author Contributions

FZ contributed to the conceptualization, methodology, formal analysis, investigation, data curation, writing—original draft, writing—review and editing, and visualization. XL contributed to the investigation, formal analysis, data curation, and writing—review and editing. FC, YaW, LW, and YfW contributed to the investigation and writing—review and editing. MZ contributed to the conceptualization, methodology, formal analysis, writing—original draft, writing—review and editing, visualization, and supervision. All authors contributed to the article and approved the submitted version.

## Conflict of Interest

The authors declare that the research was conducted in the absence of any commercial or financial relationships that could be construed as a potential conflict of interest.

## Publisher’s Note

All claims expressed in this article are solely those of the authors and do not necessarily represent those of their affiliated organizations, or those of the publisher, the editors and the reviewers. Any product that may be evaluated in this article, or claim that may be made by its manufacturer, is not guaranteed or endorsed by the publisher.
